# The risk of morbidity and mortality following recurrent malaria in Papua, Indonesia: a retrospective cohort study

**DOI:** 10.1186/s12916-020-1497-0

**Published:** 2020-02-20

**Authors:** Saber Dini, Nicholas M. Douglas, Jeanne Rini Poespoprodjo, Enny Kenangalem, Paulus Sugiarto, Ian D. Plumb, Ric N. Price, Julie A. Simpson

**Affiliations:** 1 Centre for Epidemiology and Biostatistics, Melbourne School of Population and Global Health, The University of Melbourne, Melbourne, VIC Australia; 2 Global Health Division, Menzies School of Health Research and Charles Darwin University, Darwin, Northern Territory Australia; 3 Centre for Tropical Medicine and Global Health, Nuffield Department of Clinical Medicine, University of Oxford, Oxford, UK; 4 Timika Malaria Research Program, Papuan Health and Community Development Foundation, Timika, Papua Indonesia; 5 Department of Child Health, Faculty of Medicine, University Gadjah Mada, Yogyakarta, Indonesia; 6 Mimika District Health Authority, Timika, Papua Indonesia; 7 Rumah Sakit Mitra Masyarakat, Timika, Papua Indonesia; 8 Mahidol-Oxford Tropical Medicine Research Unit, Faculty of Tropical Medicine, Mahidol University, Bangkok, Thailand

**Keywords:** Malaria recurrence, Plasmodium, Vivax, Falciparum, Papua, Indonesia

## Abstract

**Background:**

An acute episode of malaria can be followed by multiple recurrent episodes either due to re-infection, recrudescence of a partially treated parasite or, in the case of *Plasmodium vivax* or *P. ovale*, relapse from the dormant liver stage of the parasite. The aim of this study was to quantify the impact of recurrent malaria episodes on morbidity and mortality in Papua, Indonesia.

**Methods:**

We undertook a retrospective analysis of routinely collected data from malaria patients attending the primary referral hospital in Papua, Indonesia, between April 2004 and December 2013. Multi-state modelling was used to estimate the effect of recurring malaria episodes on re-presentation and admission to hospital and death. The risks of early (≤ 14 days) and late (15 to 365 days) hospital admission and death were estimated separately in our study to distinguish between the direct and indirect effects of malaria recurrence on adverse outcomes.

**Results:**

A total of 68,361 patients were included in the analysis, of whom 37,168 (54.4%) presented initially with *P. falciparum*, 22,209 (32.5%) with *P. vivax*, and 8984 (13.1%) with other species. During 12 months of follow-up after each of the first four malaria episodes, 10,868 (15.9%) patients were admitted to hospital and 897 (1.3%) died. The risk of re-presenting to the hospital with malaria increased from 34.7% (95% CI 34.4%, 35.1%) at first episode to 58.6% (57.5%, 59.6%) following the third episode of malaria. After adjusting for co-factors, infection with *P. vivax* was a significant risk factor for re-presentation (hazard ratio (HR) = 1.48 (95% CI 1.44, 1.51)) and late admission to hospital (HR = 1.17 (1.11, 1.22)). Patients infected with *P. falciparum* had a greater overall rate of mortality within 14 days (HR = 1.54 (1.25, 1.92)), but after multiple episodes of malaria*,* there was a trend towards a higher rate of early death in patients infected with *P. vivax* compared to *P. falciparum* (HR = 1.91 (0.73, 4.97)).

**Conclusions:**

Compared to patients initially infected with *P. falciparum,* those infected with *P. vivax* had significantly more re-presentations to hospital with malaria, and this contributed to a high risk of inpatient admission and death. These findings highlight the importance of radical cure of *P. vivax* to eliminate the dormant liver stages that trigger relapses.

**Electronic supplementary material:**

The online version of this article (10.1186/s12916-020-1497-0) contains supplementary material, which is available to authorized users.

## Background

Malaria remains a major threat to health in malaria-endemic countries, where it is associated with significant morbidity, mortality and a high socioeconomic burden [1]. Malaria treatment protocols prioritise the mitigation of the immediate complications of infection. However, there is growing evidence of the clinical importance of recurrent episodes of malaria which are associated with cumulative morbidity and an increased risk of mortality [2]. Prevention of recurrence should therefore be an integral part of acute malaria management. Recurrent episodes of malaria can arise from either inadequate clearance of the initial blood stage infection (recrudescence) or, in patients continuing to reside in endemic settings, a new infection from a mosquito (re-infection). Unlike *P. falciparum*, *P. vivax* can form dormant liver stages (hypnozoites) which can reactivate weeks to months after the initial infection to cause further blood stage infections and clinical illness known as relapses.

The acute illness of malaria is attributable to the asexual blood stages of the parasite, which are treated by administration of blood schizonticides, such as chloroquine- or artemisinin-based therapies. Each recurrence of malaria, irrespective of whether it is a recrudescence, re-infection or a relapse, is associated with haemolysis due to rupture of both infected and uninfected red blood cells, compounded by dyserythropoesis, both of which lead to an increased risk of severe anaemia and associated morbidity and mortality [3, 4]. Primaquine, the only widely available antimalarial active against the dorman liver stages, can be used to prevent *P. vivax* relapses; however, poor adherence to the standard 14-day regimen limits its effectiveness [3] and its associated risk of haemolysis in G6PD-deficient patients makes health providers reluctant to prescribe it [5, 6].

Papua, the most easterly province of Indonesia, is co-endemic for both *P. falciparum* and *P. vivax* malaria. In this region, antimalarial drug resistance has emerged in both species, and the local population is at significant risk of recurrent malaria [6–9]. The aim of this study was to determine the impact of recurrent hospital presentations on the risks of hospital admission and death. Routinely collected data from patients who attended the Rumah Sakit Mitra Masyarakat (RSMM) with malaria between April 2004 and December 2013 were analysed, to estimate the risks of morbidity and mortality associated with multiple episodes of malaria and the demographic and clinical risk factors for admission to hospital and death.

## Methods

### Study site

The study was undertaken in Timika, the capital of Mimika District located in the southern part of Papua province in eastern Indonesia. The region includes forested lowlands, coastal areas and subalpine and alpine regions. Malaria transmission is restricted to lowland areas where rainfall is perennial and the temperature is relatively consistent and warm [10]. In 2013, the point prevalence of parasitaemia by microscopy in Timika was 16.3%; 46% of which was due to *P. falciparum*, 39% *P. vivax*, 4% *P. malariae* and 11% mixed infections [11]. *Plasmodium ovale* infections are rare. Local *P. vivax* strains have a typical equatorial relapse periodicity of 3–4 weeks.

The population of Mimika District was estimated at 120,457 in 2004 rising to 189,447 in 2013, mostly comprising Highland and Lowland Papuans of Melanesian ancestry as well as Indonesians from elsewhere in the country.

The RSMM is a hospital with a busy outpatient department, emergency department and 110 inpatient beds. Until January 2010, it was the only public referral hospital in Mimika District. Hospital administrators collect demographic data, clinical information (including ICD10 codes assigned by the attending physician) and vital status information for each patient presentation and link each record to a unique hospital identification number. Data from the hospital pharmacy and the hospital’s full blood coulter counter are also collected and linked to the same individual identification number. Hospital protocol dictates that all inpatients and any outpatient with symptoms potentially consistent with malaria have a blood sample taken for malaria microscopy and/or a rapid diagnostic test.

Before 2006, oral quinine was the first-line treatment for falciparum malaria in the hospital with a 14-day course of primaquine added for patients with vivax malaria [12]. After a change in antimalarial policy in March 2006, the first-line treatment for uncomplicated malaria, due to any *Plasmodium* species was changed to dihydroartemisinin-piperaquine (DHP) plus 14 days of unsupervised primaquine (total dose 7 mg/kg) for patients with vivax malaria. At the same time, the first-line treatment of severe malaria was changed from intravenous quinine to intravenous artesunate [10].

### Study design

This was a retrospective analysis of routinely collected data including all patients older than 1 month who presented to RSMM between April 2004 and December 2013 at least once with malaria due to any *Plasmodium* species. Patients younger than 1 month were excluded to avoid the confounding effects of congenital infection and perinatal mortality [13]. The hospital administrators allocated each patient a unique identification number, from which patients could be tracked through multiple outpatient or inpatient clinical encounters. The primary outcomes of interest were re-presentation with malaria, admission for inpatient treatment (for any reason) and all-cause mortality. All patients were presumed to be at risk of either re-presentation, hospital admission or death until 12 months after the start of their current episode (1 to 4) of malaria or 31 of December 2013, whichever occurred first; hence, the maximum total duration of follow-up was 48 months. The primary explanatory variable of interest was the *Plasmodium* species at each malaria episode. Other potential confounders included in the analysis were age, ethnicity (Highland Papuan, Lowland Papuan or non-Papuan) and sex.

### Statistical analysis

**Fig. 1:**
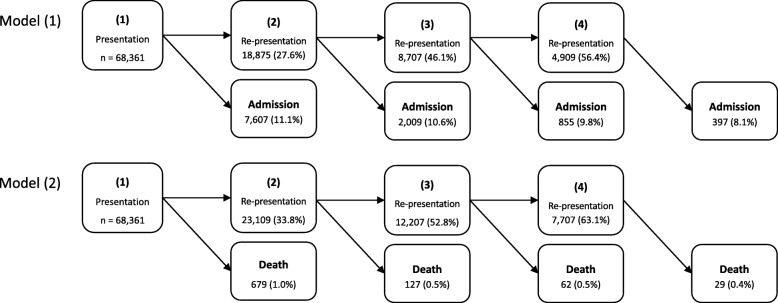
Schematic of the multi-state model. A total of 68,361 patients infected with malaria enter the study at state (1). Two separate models with different terminal states are analysed. In Model (1), the terminal state is first (hospital inpatient) admission and in Model (2) the terminal state is death. The patients start at their first malaria presentation (recorded between 2004 and 2013), and then either re-present with a malaria episode, are admitted to hospital due to any cause, die or are censored 12 months following their previous episode. Thus, at each malaria episode, a patient is at risk of re-presentation to hospital with a malaria infection or admission/death. Note, the re-presentations in Model (1) are those with only outpatient treatment as the terminal state is all-cause (hospital inpatient) admission, whilst in Model (2) each re-presentation can be accompanied with either outpatient or inpatient treatment for malaria; for more information about the multi-state model, see Section A of the Additional file 1. Frequency of re-presentations, admissions and deaths following each episode are shown; the number inside the brackets for an episode is the percentage of patients in the preceding episode who transitioned to the current episode

The impact of multiple recurrences on adverse outcomes (re-presentation, admission or death) was addressed using multi-state modelling [14–16] to quantify the transitions between malaria episodes. Multi-state modelling allows risk factors and baseline hazards to differ between malaria episodes (Section A of Additional file 1). The schematic of the multi-state models used here for modelling the transient states of malaria episodes and the terminal states is presented in Fig. 1. Two multi-state models were analysed separately. The terminal state for Model (1) was the first all-cause hospital admission and for Model (2) was death due to any cause. Patients’ hospital encounter started at their first malaria presentation. They then either re-presented with a malaria episode, were admitted to the hospital due to any cause (Model (1)), died (Model (2)) or were censored 12 months following their initial episode; all the recorded deaths were those that occurred in the hospital. Following a re-presentation, similar events to those after the initial presentation could occur within the 12 follow-up months since the re-presentation. In Model (2), re-presentations due to malaria correspond to inpatient or outpatient malaria treatments, but in Model (1), the re-presentations correspond only to outpatient treatments. Since the majority of patients (92.2%) had four or fewer episodes and a very low proportion of total deaths (6.3%) occurred following the fifth episode, the number of episodes was limited to four. A recurrence following the fourth episode was treated as a competing risk for admission/death, to ensure the validity of the results. Additional multi-state modelling was also conducted, in which state 1 of the model denoted patients admitted at their first presentation and states 2, 3, and 4 denoted re-admission (re-presentations during which inpatient care was necessary) instead of re-presentation (see Section F of Additional file 1 for further information).

The cumulative probability of re-presentation, hospital admission and death following each of the four episodes was estimated from the multi-state models and, throughout this article, is referred to as the risk of these outcomes. Cox proportional hazard modelling was used to estimate the rates of transitioning from each malaria episode to another re-presentation or admission/death and distinct baseline hazards were considered for each transition. All of the reported HRs in this manuscript are estimated using the multivariable model, adjusting for the effect of potential confounders. The results of the univariable analyses are presented in Section C of Additional file 1. To distinguish the acute/direct and indirect risks of hospital admission and death due to malaria, risks of these outcomes were estimated separately over different follow-up periods. Hospital admissions and deaths occurring within 14 days of an episode were defined as *early* hospital admissions or death. Patients who survived the initial 14-day period were considered to be at risk of *late* hospital admission and death. To determine the overall effect of the risk factors, the model was fitted initially to the data assuming that the risk factors for re-presentation and admission/death had the same effect across the multiple re-presentations. The transition-specific effects of risk factors are presented in Section B of Additional file 1. The profiles of re-presentation to hospital were very similar in Models (1) and (2); hence, for brevity, we only present the results of re-presentation for Model (1) in most cases.

The proportional hazards assumption was tested by visual inspection of the cumulative hazards. The Cox regression analyses were stratified by year to account for effect modification over the study period, due to the changing efficacy of prescribed antimalarial treatment. Since all episodes of malaria within 15 days of an initial infection were likely to reflect the acute illness rather than recurrence, the dataset for Model (1) was concatenated so that all outpatient re-presentations with malaria within this period were counted as a single event. For Model (2), the 15-day concatenation was performed on any re-presentation with malaria due to either the outpatient or inpatient departments. All statistical analyses were performed using R, version 3.5.2 [17], and the *mstate* [18] and *survival* [19] packages were used to implement and analyse the multi-state model.

## Results

During the study period, there were a total of 1,054,674 clinical presentations to the RSMM hospital, generated by 162,966 individuals. In total, 68,361 patients older than 1 month presented at least once with malaria. At the first presentation, 37,168 (54.4%) of the infections were attributable to *P. falciparum,* 22,209 (32.5%) to *P. vivax* and 7234 (10.6%) to mixed infections. *Plasmodium malariae* and *P. ovale* accounted for 1712 (2.5%) and 38 (0.1%) episodes respectively; Table 1.

**Table 1:** Baseline characteristics of patients at their first presentation with malaria to RSMM hospital, stratified by Plasmodium species (*n* = 68,361)

	Species				
	*P. falciparum*	*P. vivax*	Mix	*P. malariae*	*P. ovale*
	(*n* = 37,168)	(*n* = 22,209)	(*n* = 7234)	(*n* = 1712)	(*n* = 38)
Age, *n* (%)					
Median year (IQR^*^)	20 (6.4–28.8)	9 (2.3–23.1)	16 (3.4–25.5)	21.5 (10.4–30)	21.2 (17–26.5)
≤ 1 year	1636 (4.4)	3013 (13.6)	582 (8.0)	62 (3.6)	2 (5.3)
1–5 years	4548 (12.2)	4486 (20.2)	1245 (17.2)	141 (8.2)	1 (2.6)
5–15 years	5841 (15.7)	3189 (14.4)	1151 (15.9)	268 (15.7)	4 (10.5)
> 15 years	25,143 (67.6)	11,521 (51.9)	4256 (58.8)	1241 (72.5)	31 (81.6)
Sex, *n* (%)					
Female	17,387 (46.8)	10,776 (48.5)	3291 (45.5)	754 (44.0)	24 (63.2)
Male	19,781 (53.2)	11,433 (51.5)	3943 (54.5)	958 (56.0)	14 (36.8)
Ethnicity^†^, *n* (%)					
Non-Papuan	5364 (14.5)	4193 (18.9)	689 (9.5)	110 (6.4)	4 (10.5)
Highland	26,754 (72.1)	15,816 (71.3)	5795 (80.2)	1338 (78.2)	33 (86.8)
Lowland	4987 (13.4)	2166 (9.8)	744 (10.3)	264 (15.4)	1 (2.6)

*IQR: interquartile range (25th–75th percentiles)

†Data missing for 103 patients

### Distribution of malaria episodes

The schematic of the multi-state model and the number (and percentage) of patients re-presenting with malaria, requiring hospital admission (due to any cause) and dying within 12 months of each malaria episode are given in Fig. 1. As detailed in “Methods” section, two models were analysed separately for events following the first malaria presentation to hospital: Model (1) where the terminal event is the first admission due to any cause accompanied by an inpatient treatment (hereafter called *admission*); Model (2) where the terminal event is death due to any cause. The re-presentations in Model (1) are only those hospital attendances in which outpatients received antimalarial treatments. However, in Model (2), re-presentation could be either patients receiving antimalarial treatment in the outpatient clinic or hospital wards. Figure 1 shows that, overall, the percentage of patients re-presenting to hospital with malaria increased with each episode of malaria, whereas the percentage of patients requiring admission or dying within 12 months due to any cause decreased with each subsequent episode.

**Fig. 2:**
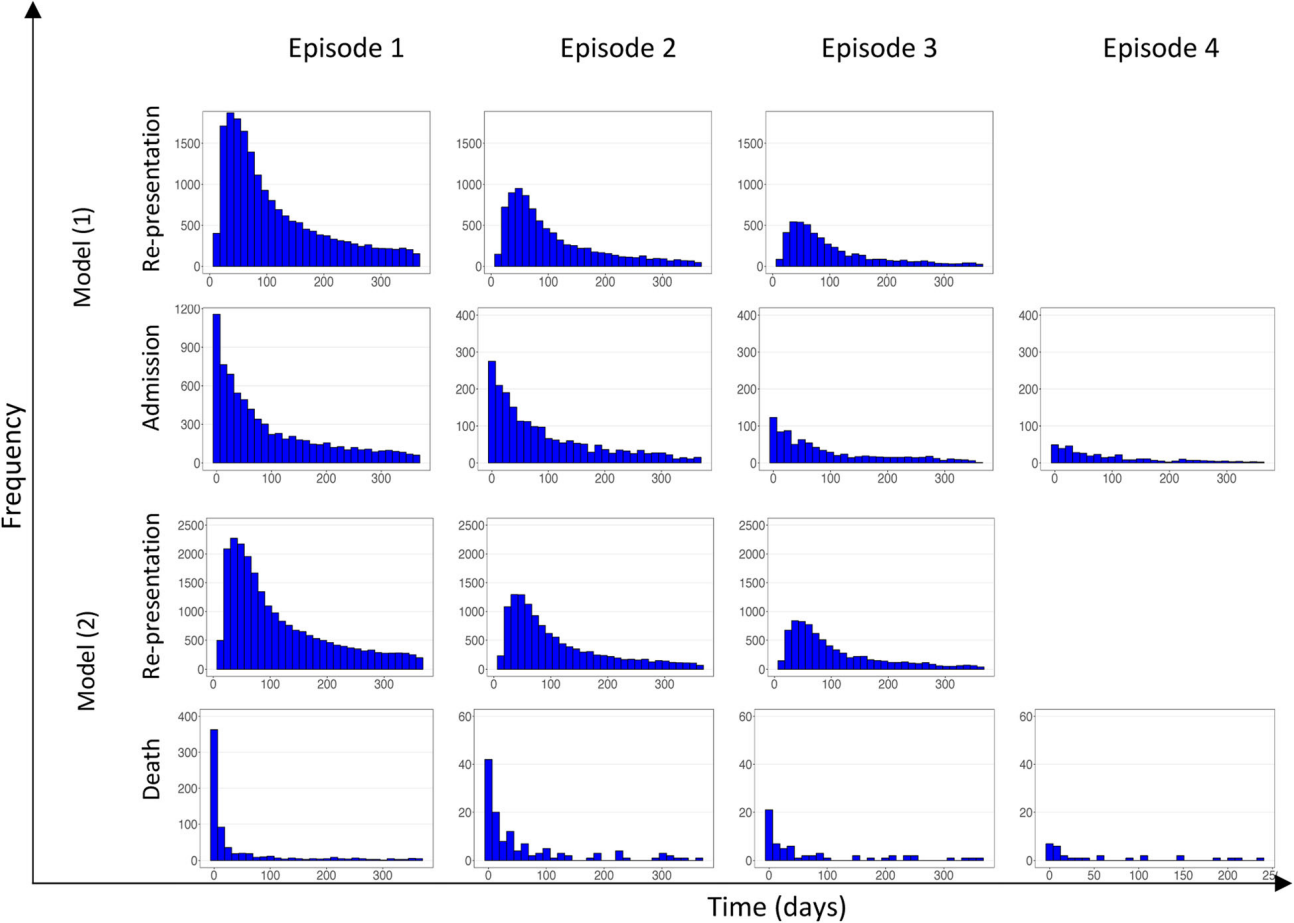
Distribution of time to event following an initial malaria infection (episode 1) or re-presentation (episodes 2 to 4). The first and second rows correspond to {1 → 2, 2 → 3, 3 → 4} and {1 → admission, 2 → admission, 3 → admission, 4 → admission}, respectively in Model (1). The third and fourth rows correspond to the same transitions as the above rows but for Model (2) where the terminal state is death. The columns from left to right correspond to the episodes 1 to 4

The times to re-presentation with malaria and all-cause admission to hospital in Model (1) and the times to re-presentation with malaria and all-cause deaths in Model (2) are presented in Fig. 2. The time to re-presentation with malaria had a log-normal distribution with a median of 82 days across all of the episodes, whereas the time to hospital admission had an exponential distribution with a fast decay rate, demonstrating that a large proportion of admissions occurred shortly after (re)presentation. Time to death followed a similar distribution as that for admission, but the decay rate was even faster (49.4% of deaths occurred within 14 days) with a smaller spread across the subsequent 12-month follow-up.

**Fig. 3:**
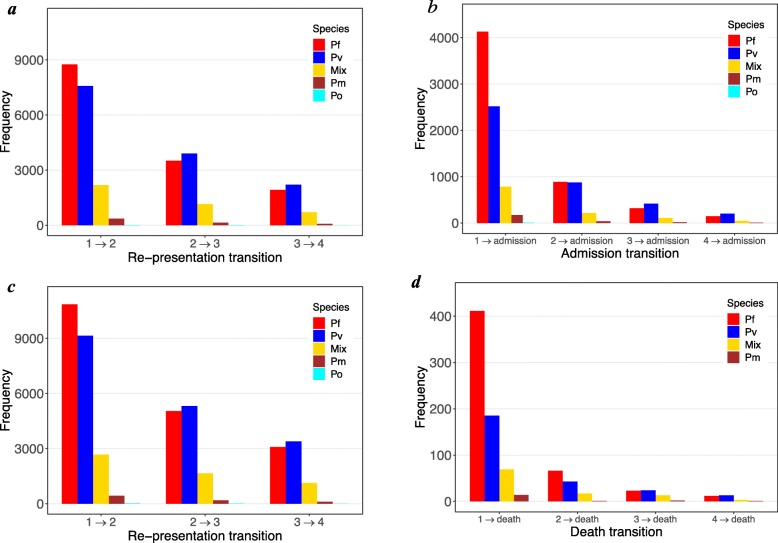
Frequency of events stratified by species. The events (re-presentation with any species, admission and death) are stratified by species at each prior episode (i.e., species at episode *j* for *j → j* + 1 transition). Top row: frequency of **a** malaria re-presentation and **b** hospital admission in Model (1). Bottom row: frequency of **c** malaria re-presentation and **d** death in Model (2). Pf—*P. falciparum*, Pv—*P. vivax*, Mix—mixed infection, Pm—*P. malariae*, Po—*P. ovale*

The frequencies of re-presentation, admission and death events stratified by species are illustrated in Fig. 3. *Plasmodium falciparum* was the most prevalent species at the first presentation in patients who re-presented to hospital, comprising 46.4% (8755/18,875; Model (1)) of transitions over episode 1 → episode 2. Thereafter, mono-infection with *P. vivax* was the main cause of re-presentation (Fig. 3a).

Following the first and second episode of malaria, *P. falciparum* was the most common species associated with admission to hospital (54.3% (4129/7607) and 44.0% (885/2009), respectively). However, after the third and fourth episodes of malaria, *P. vivax* prevailed over other species (48.5% (415/855) and 50.4% (200/397), respectively) (Fig. 3b). The pattern of *Plasmodium* species attribution was similar in Model (2), for re-presentation and death (Fig. 3c, d). Further investigations showed that 53.0% of the presentations with *P. falciparum* at the 4th episode followed at least two previous (re)presentations with *P. falciparum*; for *P. vivax*, the percentage was 56.4%*.* By counting the mixed infections at episodes 1–3 as either *P. falciparum* or *P. vivax*, these percentages rise to 63.0% and 70.3% for *P. falciparum* and *P. vivax*, respectively.

### Risk of re-presentation to hospital with malaria infection

**Fig. 4:**
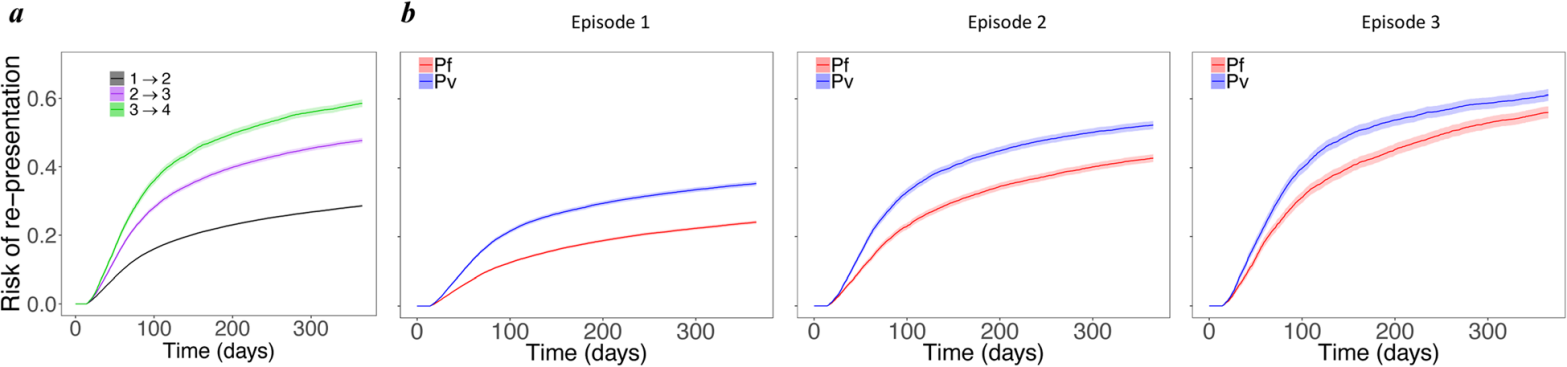
Risk of re-presentation to hospital. Cumulative probability of re-presentation for **a** all patients from episodes 1–3 and **b** only those infected with *P. falciparum* (red) and *P. vivax* (blue) species at each episode; the second to fourth columns correspond to episodes 1–3 and present the risk of re-presentation for the subsequent episode (i.e., transitions 1 → 2, 2 → 3, 3 → 4). Since the results of re-presentation were almost identical in Models (1) and (2), only the results of Model (1) are shown

The risk of re-presentation to hospital with malaria at 12 months rose from 34.7% (95% CI 34.4, 35.1) following the first episode to 58.6% (57.5, 59.6) following the 3rd episode (Fig. 4a). The risk of re-presentation for patients infected with *P. vivax* was significantly higher compared to *P. falciparum* across all of the episodes (Fig. 4b).

**Fig. 5:**
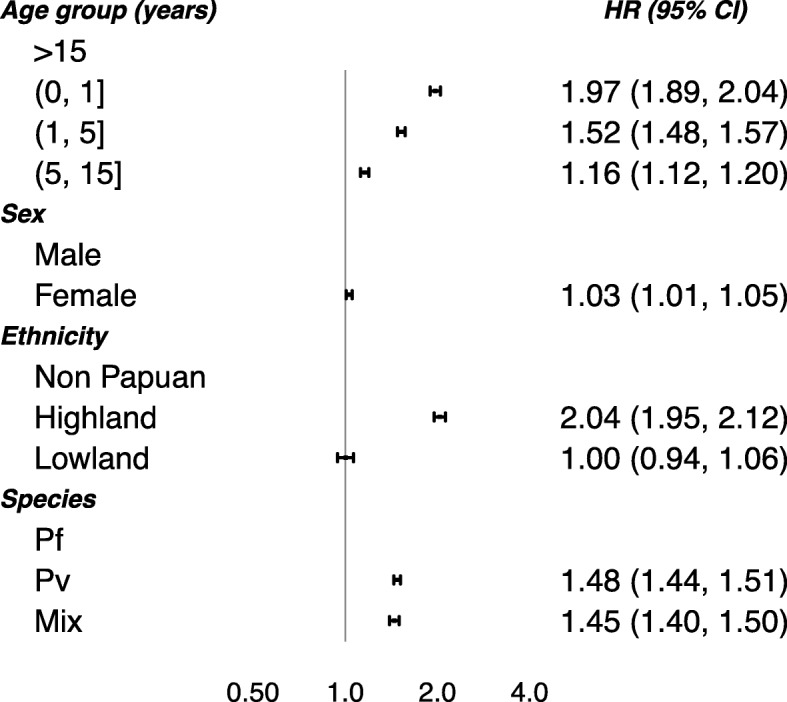
Risk factors of re-presentation to hospital. Adjusted hazard ratios (HRs; 95% confidence interval) of the associations between the age, sex, ethnicity and malaria species (Pf—*P. falciparum*, Pv—*P. vivax*, Mix—mixed infection), and any re-presentation with malaria. The HRs of re-presentation in Model (2) are not shown for brevity, because the values were very similar to Model (1) estimates. The risk factors were considered to have the same effect across the re-presentation transitions. The patients with *P. malariae* and *P. ovale* infections were excluded from the analysis due to rare number of events. The age categories (0, 1], (1, 5] and (5, 15] represent the ages >0 to ≤1 years, >1 to ≤ 5 years and >5 to ≤15 years, respectively

The demographic and clinical risk factors of re-presentation obtained from the multivariable model are shown in Fig. 5. The rate of re-presentation to hospital declined with age. Compared to adults, the hazard ratio (HR) for re-presentation was 1.97 (95% CI 1.89, 2.04) in infants (≤ 1 year), 1.52 (1.48, 1.57) in young children (1–5 years old), and 1.16 (1.12, 1.20) in older children (5–15 years). The rate of re-presentation was greater in highland Papuans compared to non-Papuans (HR = 2.04 (1.95, 2.12)) and slightly increased in females compared to males (HR = 1.03 (1.01, 1.05)). After adjustment for these risk factors, the rate of re-presentation with *P. vivax* was still significantly higher than that in patients with *P. falciparum* (HR = 1.48 (1.44, 1.51)). Patients with mixed infection also had a higher rate of re-presentation to hospital than those infected with *P. falciparum* (HR = 1.45 (1.40, 1.50)) (Fig. 5).

### Risk of all-cause hospital admission following recurrent episodes of malaria

A total of 10,868 patients were admitted to hospital following either their initial or subsequent episodes of malaria. Overall, 5381 (49.5%) of admissions were due to malaria, and 1696 (15.6%) admissions occurred within 14 days of the initial malaria episode.

**Fig. 6:**
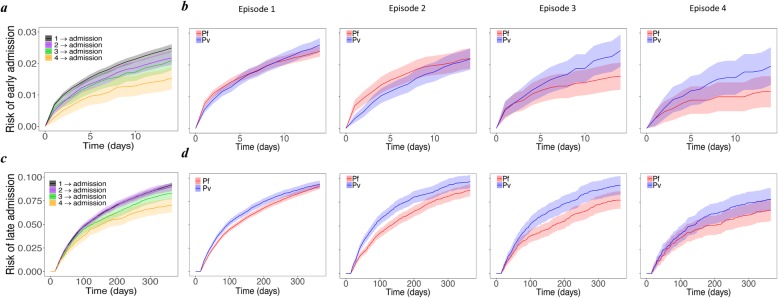
Risk of all-cause admission to hospital. Cumulative probability of early (top row) and late (bottom row) all-cause admission to hospital **a**, **c** for all patients from episodes 1–4 and **b**, **d** only for those infected with *P. falciparum* (red) and *P. vivax* (blue); the second to fifth columns correspond to episodes 1–4, respectively. Early and late admissions are defined as the first hospital admission (receiving inpatient treatment) within 14 days and between 15 and 365 days of an episode, respectively

The risk of all-cause admission to hospital within 14 days was 2.49% (95% CI 2.37, 2.61) after the first presentation with malaria, but fell to 1.53% (1.18, 1.88) after the 4th episode (Fig. 6a). For patients infected with *P. falciparum*, the risk of early admission decreased significantly with malaria recurrence, but this was not apparent for patients with *P. vivax*. By the third episode of re-presentation with any malaria species, the risk of early hospital admission following *P. vivax* infection was 2.45% (1.94, 2.95) compared to 1.64% (1.21, 2.07) following *P. falciparum* (Fig. 6b).

The risks of late admission (after day 14 and at 12 months) were similar, 9.19% (95%CI 8.96, 9.42) following the first episode and 8.98% (8.56, 9.41) following the second episode, but declined thereafter, falling to 7.02% (6.27, 7.77) after the fourth episode (Fig. 6c). Patients with *P. vivax* were at a greater risk of late admission compared to *P. falciparum,* and this was apparent following most episodes (Fig. 6d).

**Fig. 7:**
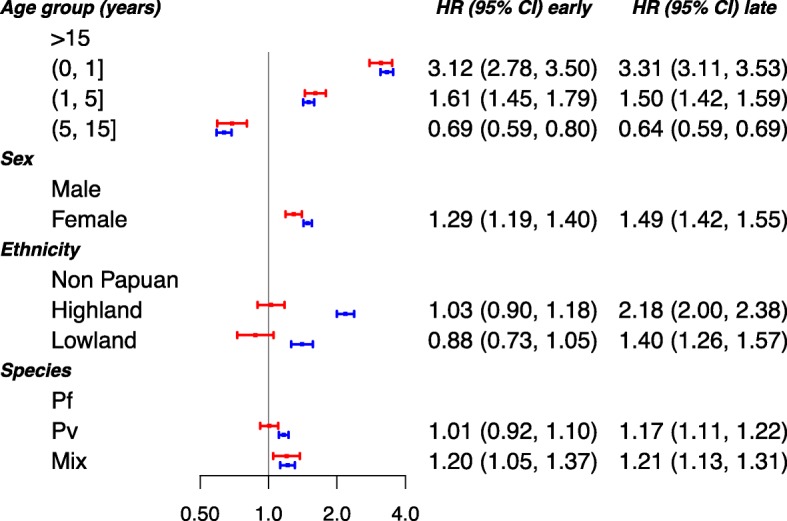
Risk factors of all-cause admission to hospital. Adjusted hazard ratios (HRs; 95% confidence interval) of the associations between the age, sex, ethnicity and malaria species (Pf—*P. falciparum*, Pv—*P. vivax*, Mix—mixed infection), and all-cause admission to hospital. The risk factors were considered to have the same effect across the admission transitions. The patients with *P. malariae* and *P. ovale* infections were excluded from the analysis due to rare number of events. The red and blue solid circles correspond to estimates of HR for early and late admission, respectively. The age categories (0, 1], (1, 5] and (5, 15] represent the ages >0 to ≤1 years, >1 to ≤ 5 years and >5 to ≤15 years, respectively

Compared to adults, infants less than 1 year old were at the greatest risk of early (HR = 3.12 (95% CI 2.78, 3.50)) and late (HR = 3.31 (3.11, 3.53)) hospital admission (Fig. 7). The rates of both early and late hospital admission were also higher in females: HR = 1.29 (1.19, 1.40) and 1.49 (1.42, 1.55) respectively. Compared to non-Papuans, highland and lowland Papuans were at greater risk of later admission to hospital (HR = 2.18 (2.00, 2.38) and 1.40 (1.26, 1.57) respectively), but there was no difference in the rate of early admission to hospital. The rate of late admission to hospital was greater following *P. vivax* than *P. falciparum* infections (HR = 1.17 (1.11, 1.22)); both rates of early and late admission (HR = 1.20 (1.05, 1.37) and 1.21 (1.13, 1.31) respectively) were greater after mixed infections compared with *P. falciparum*. Furthermore, the hazard ratio for the rate of late admissions following *P. vivax* infection compared to *P. falciparum* was highest in young children, with an HR of 1.25 (1.11, 1.42) in infants less than 1 year old and 1.36 (1.23, 1.50) in children aged 1 to 5 years. The corresponding HRs were 1.06 (0.90, 1.24) in older children (5 to 15 years old), and 1.09 (1.02, 1.17) in adults older than 15 years (Section D of the Additional file 1).

### Risk of all-cause death following recurrent episodes of malaria

**Fig. 8:**
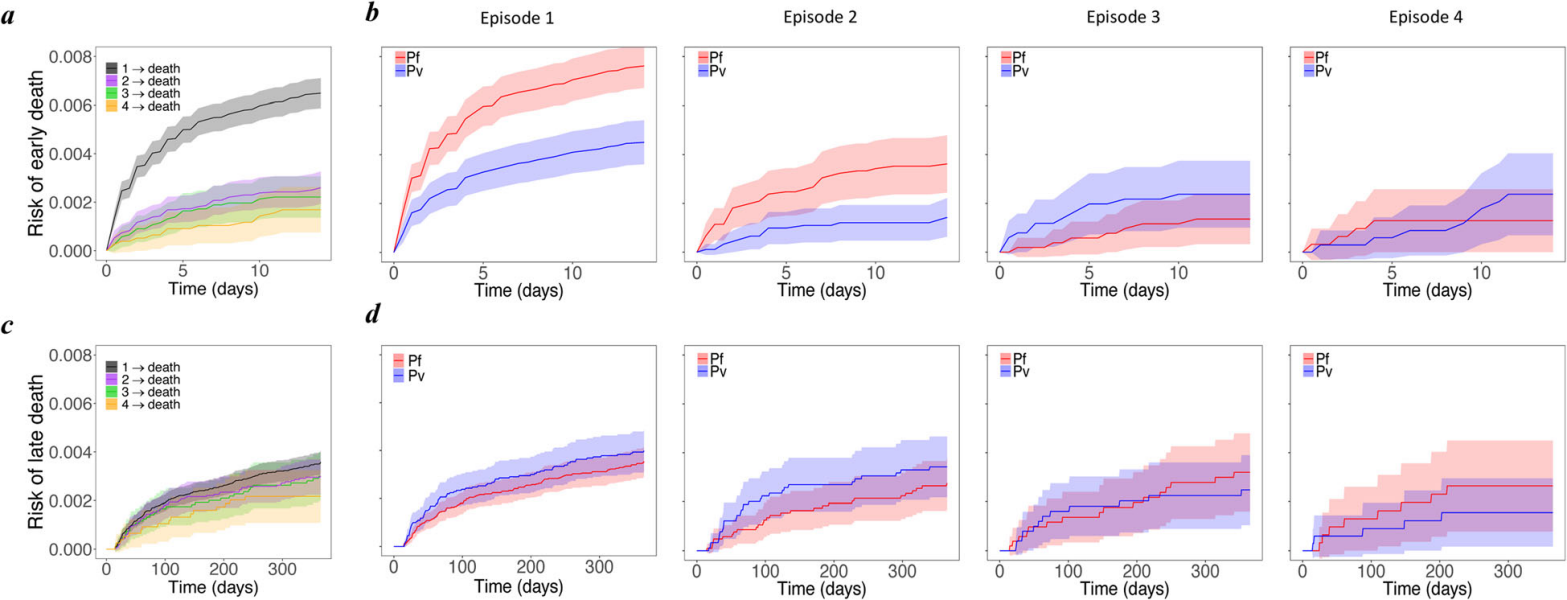
Risk of all-cause death. Cumulative probability of early (top row) and late (bottom row) death **a**, **c** for all patients from episodes 1–4 and **b**, **d** only for those infected with *P. falciparum* (red) and *P. vivax* (blue); the second to fifth columns correspond to episodes 1–4, respectively. Early and late death are defined as the deaths within 14 days and between 15 and 365 days of an episode, respectively

A total of 897 (1.3%) patients died with 75.7% (679) of deaths occurring following the first episode of malaria. The risk of early death (within 14 days) was 0.65% (95%CI 0.59, 0.71) after the first episode of malaria, but fell thereafter (Fig. 8a). When the early deaths were excluded, the risk of death within 12 months of following the first episode of malaria fell to 0.36% (0.31, 0.40) (Fig. 8c). In patients infected with *P. falciparum,* the risk of early death was 0.76% (0.67, 0.85) following the first episode of malaria and 0.36% (0.24, 0.48) following the second episode. These risks were significantly lower in patients initially infected with *P. vivax* infection (0.45% (0.36, 0.54) and 0.14% (0.06, 0.22) respectively)*.* However, for subsequent episodes, this trend was inverted, the risk of death rising to 0.24% (0.10, 0.37)) after the third episode of *P. vivax* compared to 0.14% (0.03, 0.24) following the third episode of *P. falciparum* (Fig. 8b). In the episode-specific multivariable model (see Section B of Additional file 1), after controlling for confounding factors, there was a trend for a higher rate of early death with *P. vivax* infection compared to *P. falciparum* after the third episode (HR = 1.91 (0.73, 4.97)). Similarly, mixed infections were associated with a higher rate of early death after three episodes of malaria, compared to *P. falciparum* (HR = 3.68 (1.27, 4.18)) (Section B of Additional file 1).

**Fig. 9:**
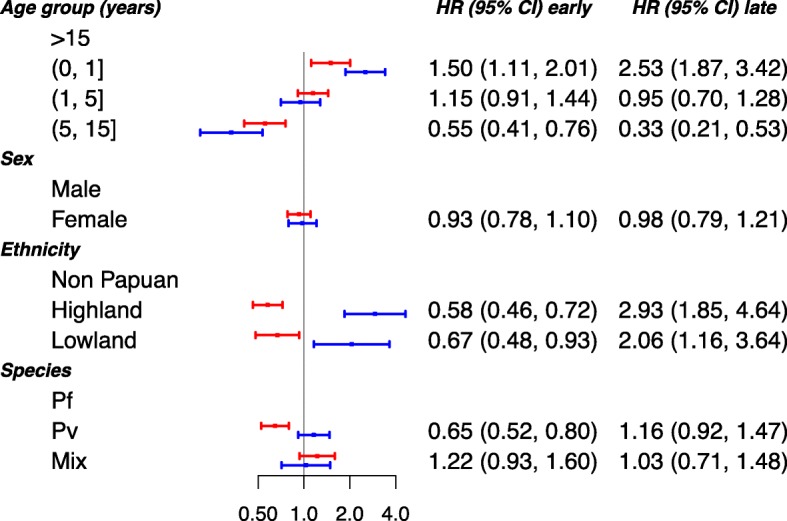
Risk factors of all-cause death. Adjusted hazard ratios (HRs; 95% confidence interval) of the associations between the age, sex, ethnicity and malaria species (Pf—*P. falciparum*, Pv—*P. vivax*, Mix—mixed infection), and death. The risk factors of death were considered to have the same effect across the transitions. The patients with *P. malariae* and *P. ovale* infections were excluded from the analysis due to rare number of events. The red and blue solid circles correspond to estimates of HR for early and late death, respectively. The age categories (0, 1], (1, 5] and (5, 15] represent the ages >0 to ≤1 years, >1 to ≤ 5 years and >5 to ≤15 years, respectively

The overall rate of early death following *P. vivax* was 0.65-fold (95% CI 0.52, 0.80) that of *P. falciparum* (or alternatively, *P. falciparum* had an increased rate of early death of 1.54-fold (1.25, 1.92) compared to *P. vivax*), whereas for late death this rose to 1.16-fold (0.92, 1.47) (Fig. 9).

Severity of the initial episode significantly impacted the rates of early and late death (Section E of Additional file 1). Compared to patients treated as outpatients, those requiring admission on the first episode had higher rates of early and late death for both of the species; the HRs for *P. falciparum* were 9.54 (7.35, 12.37) and 1.84 (1.36, 2.50), respectively, and those for *P. vivax* were 10.26 (7.21, 14.60) and 2.58 (1.75, 3.81), respectively.

## Discussion

Previous studies have demonstrated that recurrent episodes of malaria are associated with an increased cumulative risk of anaemia, malnutrition and sepsis [9, 20, 21]. To explore the relationship between multiple episodes of malaria and morbidity and mortality, we used multi-state modelling to investigate how recurring episodes of malaria influence the risks of re-presentation to hospital, all-cause hospital admission and all-cause death following an initial presentation to hospital with malaria infection in Papua, Indonesia. Particular attention was paid to how the comparative risks of morbidity and mortality attributable to *P. vivax* and *P. falciparum* changed across multiple malaria episodes.

Patients initially presenting with *P. vivax* infection were 1.5-fold more likely to re-present with malaria than patients initially infected with *P. falciparum,* a reflection of *P. vivax’s* ability to relapse weeks to months following an initial infection [22]. The blood stage infections of all *Plasmodium* species were treated with the same schizontocidal regimens (quinine before March 2006 and dihydroartemisinin-piperaquine after March 2006). Patients with *P. vivax* were also offered radical cure with 14 days of primaquine to eradicate the dormant liver stages, but previous studies in this population have shown that when unsupervised this regimen is associated with very poor effectiveness [3]. Furthermore, many of the malaria re-presentations following initial infection with *P. falciparum* will also have been attributable to *P. vivax*, since in co-endemic areas, there is a high risk of heterologous *P. vivax* relapse following falciparum malaria [23–27].

Overall, patients were significantly more likely to have a late admission or late death respectively following a *P. vivax* infection compared to a *P. falciparum* infection, and this remained apparent after controlling for baseline characteristics such as age*.* We hypothesise that the cumulative risk of anaemia attributable to recurrent bouts of malarial haemolysis and dyserythropoiesis underlies the higher risks of adverse outcomes after repeated *P. vivax* infections. Indeed, in this region the haematological morbidity due to *P. vivax* malaria is particularly severe [28–30]. Although acute infection with *P. falciparum* results in a more severe acute disease than *P. vivax*, multiple re-presentations with vivax malaria may cause either a debilitating illness [31] or may arise in individuals with severe comorbidities that render the patient more susceptible to severe disease and death. The former plays an important role in both the direct and indirect mortality of *P. vivax*. In a cohort of children from Vanuatu, infection with *P. vivax*, but not *P. falciparum*, was a major predictor of acute malnutrition [32], and our previous analyses in Papua Indonesia have shown that malnourished children with *P. vivax* are at high risk of both acute and delayed mortality [2, 9, 21].

In our current analysis, the species causing malaria exerted varying effects on morbidity and mortality across multiple malaria episodes. *P. falciparum* malaria was the major species causing early admission and death following the first two malaria episodes. However, after two re-presentations with malaria, this trend was inverted such that the cumulative risks of early admission and death with *P. vivax* infection rose to 1.5- and 1.7-fold, respectively, higher than the risks following *P. falciparum* malaria*.* Similar higher risks were observed following more than two re-presentations with *P. vivax*.

In southern Papua, Indonesia, the proportion of malaria morbidity and mortality attributable to *P. vivax* infection has increased over the last 20 years [10]. The proportion of malaria cases due to *P. vivax* at RSMM rose from 32% in 2004 to 54% by 2009. Whereas the risk of death due to *P. falciparum* over the same time period halved, the proportion of deaths due to *P. vivax* remained steady. The differential impact of malaria control activities on the two species is likely due to the inadequate radical cure of *P. vivax* and prevention of multiple relapses [3]. Young children are at particularly high risk of recurrent vivax infection and associated morbidity and mortality [9].

Our study has some important limitations. First, the data used in this work are left-truncated; hence, the patients’ history of malaria prior to the start of data collection in 2004 is unknown. This has resulted in some disparities between the risk of hospital admissions and death for the first and the subsequent episodes. For instance, the acute condition of patients (characterised by the large number of early deaths) at the first episode may denote possible frequent malaria recurrences prior to the first presentation. Distinguishing between the early and late mortality helped to mitigate this issue. Second, due to the passive follow-up of the patients, episodes of malaria treated in the community will have been missed and therefore the true number of clinical malaria recurrences experienced by the individuals in this study will have been higher than that reported. However, the attrition bias in the detection of severe episodes of malaria requiring admission to hospital or resulting in death is likely to be low, since RSMM was the primary facility providing inpatient care in the region during the study period; this was confirmed by a community household survey of treatment-seeking behaviour in 2005, in which 82% (9/11) of children who died in the preceding year were reported to have had done so at the RSMM hospital [11]. We hypothesise that any attrition bias will be similar between patients with *P. falciparum* and *P. vivax*; hence, the comparative hazards presented are likely to be valid and our estimates of mortality conservative. Third, our study focused on the effect of malaria species at an episode of interest, although consideration of the history of the species during the preceding malaria infections may have been more relevant to the outcome of the disease. However, accommodating the history of infections would require non-Markovian multi-state modelling and a much larger number of deaths to estimate precisely the risks associated with each species distribution of prior infections. Finally, the increased risk of all-cause mortality following the initial infection cannot be attributed solely to malaria. Our study does not address causality, but rather quantifies the difference between *P. vivax* and *P. falciparum* in the risk of early/late morbidity and mortality following recurrent episodes of malaria.

## Conclusions

Our results highlight that infection with *P. falciparum* is associated with a greater acute risk of severe and fatal outcome than infection with *P. vivax*. In absolute terms, most deaths and admissions related to malaria occur after the first clinical episode. However, malaria recurrence is also associated with increasingly poor outcomes particularly in infants and young children. Compared to patients initially presenting with *P. falciparum* malaria, those presenting with *P. vivax* were at a significantly greater risk of recurrent malaria, and this was associated with a higher risk of mortality. Whilst the acute management of malaria is paramount to prevent early death, our analysis highlights the importance of preventing recurrent malaria. The latter can be achieved either through bed-net distribution, chemoprophylaxis or, in the case of *P. vivax*, the delivery of safe and effective radical cure of the hypnozoite reservoir of infection.

## Supplementary information

Additional file 1. The file contains details of the developed multi-state model and some additional statistical analyses under the following sections: A. Multi-state modelling of malaria recurrence; B. Episode-specific effects of risk factors; C. Results of univariable analysis; D. Effect modification of species on the rate of events by patients’ age; E. Effect of severity of the initial episode on mortality; F. Re-admission and death multi-state model.

## Data Availability

The datasets analysed during the current study are available from the corresponding author on reasonable request.

## Abbreviations

CI: Confidence interval
CQ: Chloroquine
HR: Hazard ratio
Pf: *Plasmodium falciparum*
Pm: *Plasmodium malariae*
Po: *Plasmodium ovale*
Pv: *Plasmodium vivax*
RSMM: Rumah Sakit Mitra Masyarakat
