# Supplementary material for: The risk of morbidity and mortality following recurrent malaria in Papua, Indonesia: a retrospective cohort study
Source: BMC Med. 2020 Feb 20;18:28. doi: 10.1186/s12916-020-1497-0 (PMC7031957; doi:10.1186/s12916-020-1497-0)
Supplement: Supplementary file 1 — The file contains details of the developed multi-state model and some additional statistical analyses under the following sections: A. Multi-state modelling of malaria recurrence; B. Episode-specific effects of risk factors; C. Results of univariable analysis; D. Effect modification of species on the rate of events by patients’ age; E. Effect of severity of the initial episode on mortality; F. Re-admission and death multi-state model. [file 12916_2020_1497_MOESM1_ESM.docx]

Additional File 1

Dini *et al.*, The risk of morbidity and mortality following recurrent malaria in Papua, Indonesia: a retrospective cohort study

[A. Multi-state modelling of malaria recurrence 1](#_Toc30580885)

[B. Episode-specific effects of risk factors 4](#_Toc30580886)

[C. Results of univariable analysis 5](#_Toc30580887)

[D. Effect modification of species on the rate of events by patients’ age 6](#_Toc30580888)

[E. Effect of severity of the initial episode on mortality 6](#_Toc30580889)

[F. Re-admission and death multi-state model 7](#_Toc30580890)

1. Multi-state modelling of malaria recurrence

In multi-state modelling, the changing states of patients during the course of a disease are represented by a series of states and transition rates between the states [14, 16]. Figure A.1 depicts the schematic of the multi-state model used here for modelling the transition of patients to higher malaria episodes and to the terminal states, admission or death. The patients start at their first malaria presentation (recorded between April 2004 and December 2013), and then either re-present with a malaria episode, die or are censored 12 months following their previous episode. As shown in the schematic, the number of re-presentations was limited to three (four transient states with the first presentation), mainly because the number of admission and deaths following four or more re-presentations was small. Of note, a recurrence following the third re-presentation is treated as a competing risk for death/admission, to ensure the validity of the results.

Two models were analysed separately. In Model (1), first admission (i.e. attendance to the hospital ensued by hospitalisation and receiving inpatient treatment), and in Model (2) death, are the terminal states of the multi-state model; see Figure A.1. Thereby, the re-presentations in Model (1) correspond only to outpatient treatments, as the first inpatient treatment is considered as the terminal state, but in Model (2), the re-presentations correspond to either inpatient or outpatient treatments.

A re-presentation with malaria from episode *j* causes a transition of *j →­ j+*1, and hospital admission (death in Model (2)) from that episode can occur via the transition *j →*­ admission (*j →*­ death in Model (2)). Cox proportional hazard modelling was used to estimate the rates of these transitions:

$$\begin{matrix} & r_{i}^{j\_j+1}(t)=r_{0}^{j\_j+1}(t) \exp(\boldsymbol{\beta}\cdot\mathbf{X}_{i}), \\ & \lambda_{i}^{j\_d}(t)=\lambda_{0}^{j\_d}(t) \exp(\boldsymbol{\alpha}\cdot\mathbf{X}_{i}), \end{matrix}$$

where $r_{0}^{j\_j+1}$ and $\lambda_{0}^{j\_d}$ are the baseline hazards of recurrence and admission (death) transition rates for patient $i$, respectively, and $\boldsymbol{\beta}$ and $\boldsymbol{\alpha}$ are the logarithm of the hazard ratios for the explanatory covariates $\boldsymbol{X}_{\boldsymbol{i}}$, respectively.

Clock-reset time-scale [15] was considered for the multi-state model since we were interested in evaluating the risk of events since entering an episode rather than the time since the first presentation which is considered in the clock-forward time-scale.

**Figure A.1 Schematic of the multi-state model.**

In Model (1), the terminal state is first (hospital inpatient) admission and in Model (2) the terminal state is death; these models were fitted to the data separately. $r^{j\_j+1}$ and $\lambda^{j\_d}$ are the hazards of re-presentation with malaria (*j*→­*j*+1 episode) and admission/death (*j*→­admission / *j*→­death) transitions, respectively.

1. Episode-specific effects of risk factors

b

a

c

**Figure B.1 Risk factors of re-presentation to hospital (a), hospital admission (b) and death (c), determined for each transition.**

Adjusted Hazard Ratios (HRs; 95% Confidence Interval) of the associations between the age, sex, ethnicity and malaria species (Pf *– P. falciparum,* Pv *– P. vivax,* Mix – mixed infection), and any re-presentation with malaria (panel (a)), admission (b) and death (c). The HRs of re-presentation in Model (2) are not shown for brevity, because the values were very similar to Model (1) estimates. Unlike the results shown in Figures 5, 7 and 9, the risk factors of re-presentation and admission/death were considered different across the transitions, as explained in Section A. The patients with *P. malarie* and *P. ovale* infections were excluded from the analysis due to rare number of events. The black solid circles correspond to the HRs estimated from all patients, and the red and blue solid circles correspond to estimates of HR for early and late admission/death, respectively. The HRs are undefined for some of the covariates over certain transitions due to no admission/death over these transitions. The rarity of events over some transitions (particularly those from higher episodes) has resulted in wide confidence intervals and rejection of proportional hazard assumption in these cases.

1. Results of univariable analysis

**Figure C.1 Risk factors of re-presentation to hospital, hospital admission and death estimated via the univariable Cox proportional hazard model.**

Hazard Ratios (HRs; 95% Confidence Interval) of the associations between the age, sex, ethnicity and malaria species (Pf *– P. falciparum,* Pv *– P. vivax,* Mix – mixed infection), and any re-presentation with malaria, admission and death in the univariable model. The HRs of re-presentation in Model (2) are not shown for brevity, because the values were very similar to Model (1) estimates. The risk factors of re-presentation and admission/death were considered to have the same effect across the re-presentation and admission/death transitions, respectively. The patients with *P. malarie* and *P. ovale* infections were excluded from the analysis due to rare number of events. The black solid circles correspond to the HRs estimated from all patients, and the red and blue solid circles correspond to estimates of HR for early and late admission/death, respectively. The results of the multivariable analyses are provided in the main paper, see Figures 5, 7 and 9.

1. Effect modification of species on the rate of events by patients’ age

In order to examine whether the effect of species on the rates of re-presentation, admission and death is influenced by the patients’ age, we modified the Cox regression models by adding an interaction term between age and species as follows:
$\lambda_{i}^{j\_d}(t)=\lambda_{0}^{j\_d}(t) \exp(\alpha_{1}Age + \alpha_{2}Sex + \alpha_{3}Ethnicity + \alpha_{4}Species + \alpha_{5}Age\times Species ),$
where $\alpha_{5}$ takes account of the interaction between age and species. Fitting the model to data showed that $\alpha_{5}$ for *P. vivax* is statistically significant only for the rate of late admission. The results indicate that the rate of late admission for *P. vivax* (compared to *P. falciparum*) increases for children of age lower than five (${HR}_{Age(0, 1]}= 1.25 (1.11, 1.42),$ ${HR}_{Age(1, 5]}= 1.36 (1.23 1.50$), ${HR}_{Age(5, 15]}= 1.06 (0.90, 1.24)$, ${HR}_{Age >15}= 1.09 (1.02, 1.17)$).

1. Effect of severity of the initial episode on mortality

We used receiving inpatient treatment (admission to hospital) at the first episode as a proxy for severity of a patient’s condition and included it in the Cox regression models of transition rates accordingly. Stratifying the analysis by the species at the initial episode, we found that for *P. falciparum*, receiving an inpatient treatment at the first presentation increased the rate of early and late death by 9.54-fold (7.35, 12.37) and 1.84-fold (1.36, 2.50), respectively, compared to those who received outpatient treatment. For *P. vivax,* the corresponding hazard ratios were 10.26 (7.21, 14.60) and 2.58 (1.75, 3.81), respectively.

1. Re-admission and death multi-state model

**Figure F.1 The schematic of the re-admission and death multi-state model.**

Additional multi-state modelling was conducted to study the effect of re-admissions (re-presentations during which inpatient care was necessary) to hospital on death. In this model, state 1 denotes patients admitted at their first presentation and states 2, 3 and 4 denotes re-admission instead of re-presentation.

***c***

***b***

***a***

**Figure F.2 Morbidity and mortality risks in the re-admission and death model.**

Cumulative probability of (a) re-admission from episodes 1–3, (b) early and (c) late death from episodes 1–4.

**Figure F.3 Adjusted Hazard Ratios (HRs; 95% Confidence Interval) of the associations between the age, sex, ethnicity and malaria species, and re-admissions with malaria and death.**

The HRs of re-presentation in model (2) are not shown for brevity, because the values were very similar to model (1) estimates. The risk factors of re-admission and death were considered to have the same effect across the re-admission and death transitions, respectively. The patients with *P. malarie* and *P. ovale* infections were excluded from the analysis. The black solid circles correspond to the HRs estimated from all patients, and the red and blue solid circles correspond to estimates of HR for early and late mortality, respectively.
